# Peptide-specific engagement of the activating NK cell receptor KIR2DS1

**DOI:** 10.1038/s41598-017-02449-x

**Published:** 2017-05-25

**Authors:** Anaïs Chapel, Wilfredo F. Garcia-Beltran, Angelique Hölzemer, Maja Ziegler, Sebastian Lunemann, Gloria Martrus, Marcus Altfeld

**Affiliations:** 1 Heinrich Pette Institute, Leibniz Institute for Experimental Virology, Hamburg, Germany; 2 Department of Internal Medicine, University Hospital Eppendorf (UKE), Hamburg, Germany; 3 Ragon Institute of MGH, MIT and Harvard, Cambridge, MA USA

## Abstract

The activating NK cell receptor KIR2DS1 has been shown to be involved in many disorders including autoimmune diseases, malignancies and pregnancy outcomes. However, the precise ligands and functions of this receptor remain unclear. We aimed to gain a better understanding of the factors involved in the binding of KIR2DS1 and its inhibitory counterpart KIR2DL1 to HLA class I molecules, and the consequences for KIR2DS1+ NK-cell function. A systematic screen that assessed binding to 97 HLA-I proteins confirmed that KIR2DS1-binding was narrowly restricted to HLA-C group 2 complexes, while KIR2DL1 showed a broader binding specificity. Using KIR2DS1ζ^+^ Jurkat reporter-cells and peptide-pulsed 721.221.TAP1KO-HLA-C*06:02 cells, we identified the synthetic peptide SRGPVHHLL presented by HLA-C*06:02 that strongly engaged KIR2DS1- and KIR2DL1-binding. Functional analysis showed that this HLA-C*06:02-presented peptide can furthermore activate primary KIR2DS1(+) NK cell clones. Thus, we demonstrated peptide-dependent binding of the activating NK cell receptor KIR2DS1, providing new insights into the underlying mechanisms involved in KIR2DS1-related disorders.

## Introduction

Natural killer (NK) cells play a pivotal role in containing viral replication in early stages of infection and in shaping the subsequent adaptive immune response^1^. NK cells are able to recognize and kill abnormal cells thought multiple receptors that distinguish normal host molecules, stress-induced ligands, and pathogen-associated motifs^2^. These receptors are either activating or inhibitory and constitute a fine balance of signals which tightly controls NK cell function. One of the major families of NK cell receptors, the Killer Immunoglobulin Receptors (KIRs), has been shown to impact the outcome of various diseases, in particular in association with their Human Leukocyte Antigen (HLA) class-I ligands^2–4^.

KIR family receptors are encoded by polymorphic and highly homologous genes located on human chromosome 19q13.4 within the leukocyte receptor complex (LRC)^5^. Although KIRs are characterized by an extensive number of haplotypes, they all share a similar molecular structure consisting of a type 1 transmembrane glycoprotein with ectodomains comprising either two (KIR2D) or three (KIR3D) immunoglobulin-like domains^3^. The length of the cytoplasmic tail determines whether a respective KIR is inhibitory or activating: a long cytoplasmic tail characterizes inhibitory KIRs (KIR-L) whereas a short cytoplasmic tail characterizes activating KIRs (KIR-S). Most KIRs interact with specific allotypes of HLA class I ligands^5^. In general, receptors of the KIR3D group engage HLA-A and HLA-B while KIR2D receptors interact with HLA-C molecules. HLA-C ligands can be subdivided into two groups: HLA-C group 1 (HLA-C1), characterized by an asparagine in position 80, binds to KIR2DL2 and KIR2DL3 molecules and HLA-C group 2 (HLA-C2), characterized by a lysine in position 80, preferentially binds to KIR2DL1 molecules^5^.

A growing number of studies have identified associations between the presence of the activating KIR2DS1 receptor and susceptibility to autoimmune diseases^6–8^, reproductive success^9, 10^, control of viral infections^11, 12^ and malignancy in cancer^13–15^. However, the precise ligands for KIR2DS1, and their consequences for KIR2DS1+ NK-cell function, are not well characterized. KIR2DS1 and KIR2DL1 are alleles of the same single locus and share a high degree of sequence homology in their extracellular domain^16, 17^. KIR2DS1 is distinguished by having two additional residues in the transmembrane region (Lysine 233 and Threonine 237), which interact with DAP12, an adaptor protein containing immunoreceptor tyrosine-based activation motif (ITAM)^18^. For this reason, KIR2DS1 and KIR2DL1 are generally considered as counterparts sharing the same ligand-specificity for HLA-C2 allotypes^16^. Nevertheless, crystal structure analysis of KIR2DL1 bound to HLA-C*04:01 has demonstrated that binding of KIR2DL1 is not only determined by the motifs located on the heavy chain of the HLA class I molecule but also by the sequence of the peptide presented by HLA class I^19–21^. Much less is known about the mechanisms that regulate binding of KIR2DS1 to HLA-C2^17^. It has been shown that peptides presented by the HLA-C2 molecule HLA-C*04:01 can also modulate KIR2DS1-binding^22, 23^, but the functional consequences of these interactions remain unclear. Here, we demonstrate that KIR2DS1-binding is narrowly restricted to HLA-C2 ligands while KIR2DL1 exhibited a broader HLA-C ligand specificity. Furthermore, specific HLA-C*06:02-presented peptides can modulate KIR2DS1-binding and activation of primary KIR2DS1+ NK cell clones.

## Results

### KIR2DS1 narrowly binds to HLA-C2 molecules, while KIR2DL1 has broader binding specificity for HLA class I molecules

**Figure 1:**
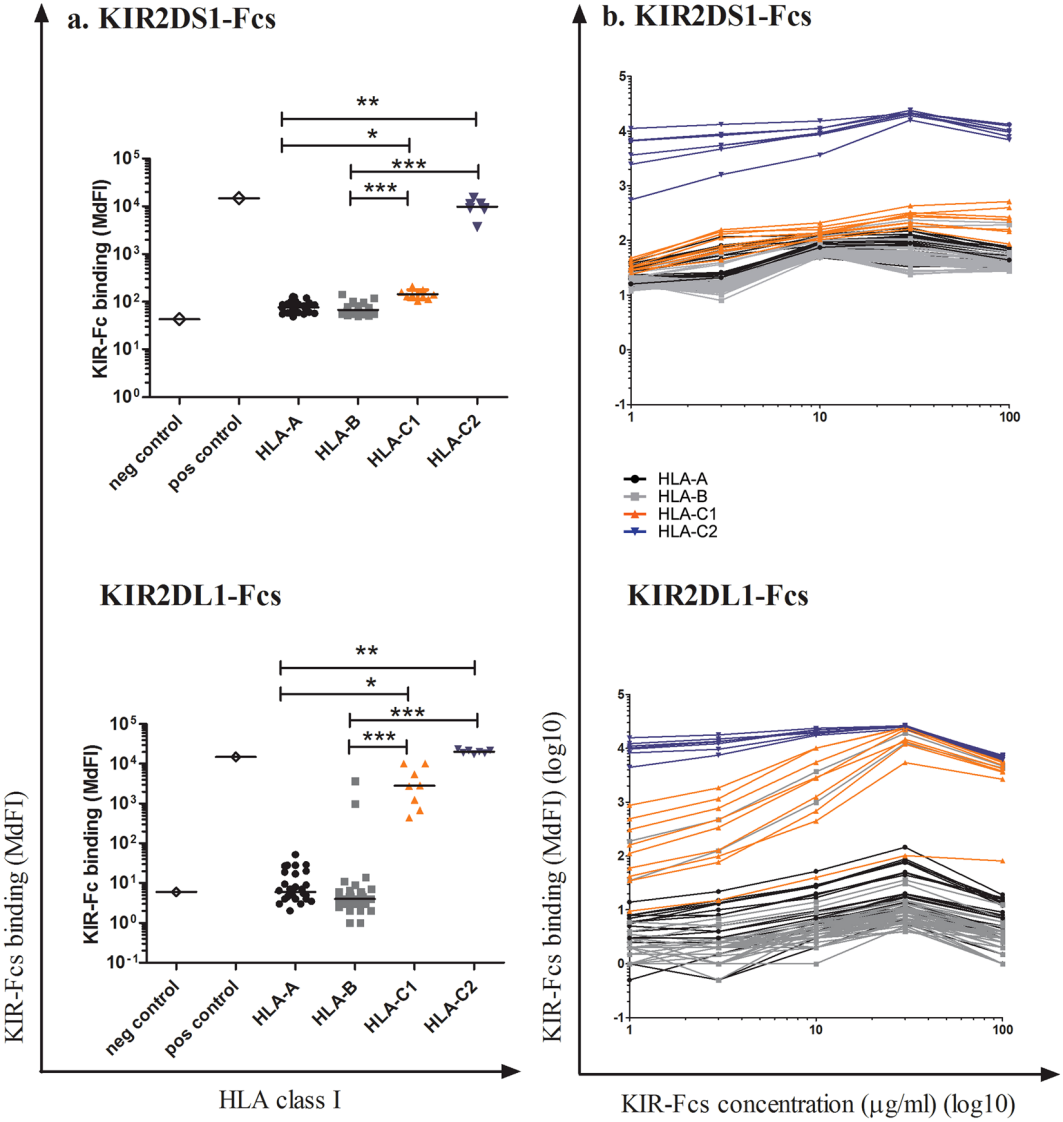
Systematic screening of KIR2DS1 and KIR2DL1 binding specificities. (**a**) KIR-Fc binding to classical HLA-I coated beads (One Lambda). KIR-Fcs were used at a concentration of 10 μg/ml. Median for each HLA-I subclasses are shown (**b**) Titration of KIR-Fcs binding to classical HLA-I coated beads (One Lambda).

A multiplex bead-based binding assay (One Lambda) consisting of 97 different beads coated with the most common allotypes of HLA-A, B, C was used to systematically assess HLA class I complex-binding to KIR2DS1- and KIR2DL1-fusion constructs (Fcs) as previously described^16, 46^ (Fig. 1). KIR2DS1 binding was exclusively restricted to HLA-C2 complexes tested (HLA-C*02:02,*04:01,*05:01,*06:02,*15:02,*17:01,*18:02), while its inhibitory counterpart KIR2DL1 showed a broader specificity (Fig. 1a) also binding to eight HLA-C1 complexes (HLA-C*01:02,*03:02,*03:03,*03:04,*07:02,*08:01,*12:03,*16:01) as well as two HLA-B complexes (HLA-B*46:01 and HLA-B*73:01). To further study the avidity of binding of HLA-I molecules to KIR2DS1 and KIR2DL1, a titration of KIR-Fcs was performed ranging from 1 to 100 μg/ml (Fig. 1b). KIR2DS1 had a high avidity for HLA-C2 complexes and reached a saturation point at 30 μg/ml. However, analysis of KIR2DL1-Fcs titrations showed two distinct binding groups with different avidity: KIR2DL1 showed high avidity binding to HLA-C2 complexes with a saturation point at 30 μg/ml, but weaker binding to the eight additional HLA-C1 and the two HLA-B complexes. The results confirmed a model in which KIR2DS1 and KIR2DL1 share the same ligand specificity for HLA-C2 complexes, but KIR2DL1 can also bind to additional HLA-C1 and HLA-B complexes with weaker affinity. Interestingly KIR2DL1-binding to the HLA-C1 complexes HLA-C*01:02, HLA-C*03:02, HLA-C*03:03, HLA-C*03:04, HLA-C*07:02, HLA-C*08:01, HLA-C*2:03 and HLA-C*16:01 was not described before, while the receptor encoded by the KIR2DL1*022 allele has already been reported to bind to HLA-B*46:01 and HLA-B*73:01^24^.

### KIR2DS1ζ^+^ and KIR2DL1ζ^+^ Jurkat reporter cells are activated by 721.221 cells expressing HLA-C2 molecules

**Figure 2:**
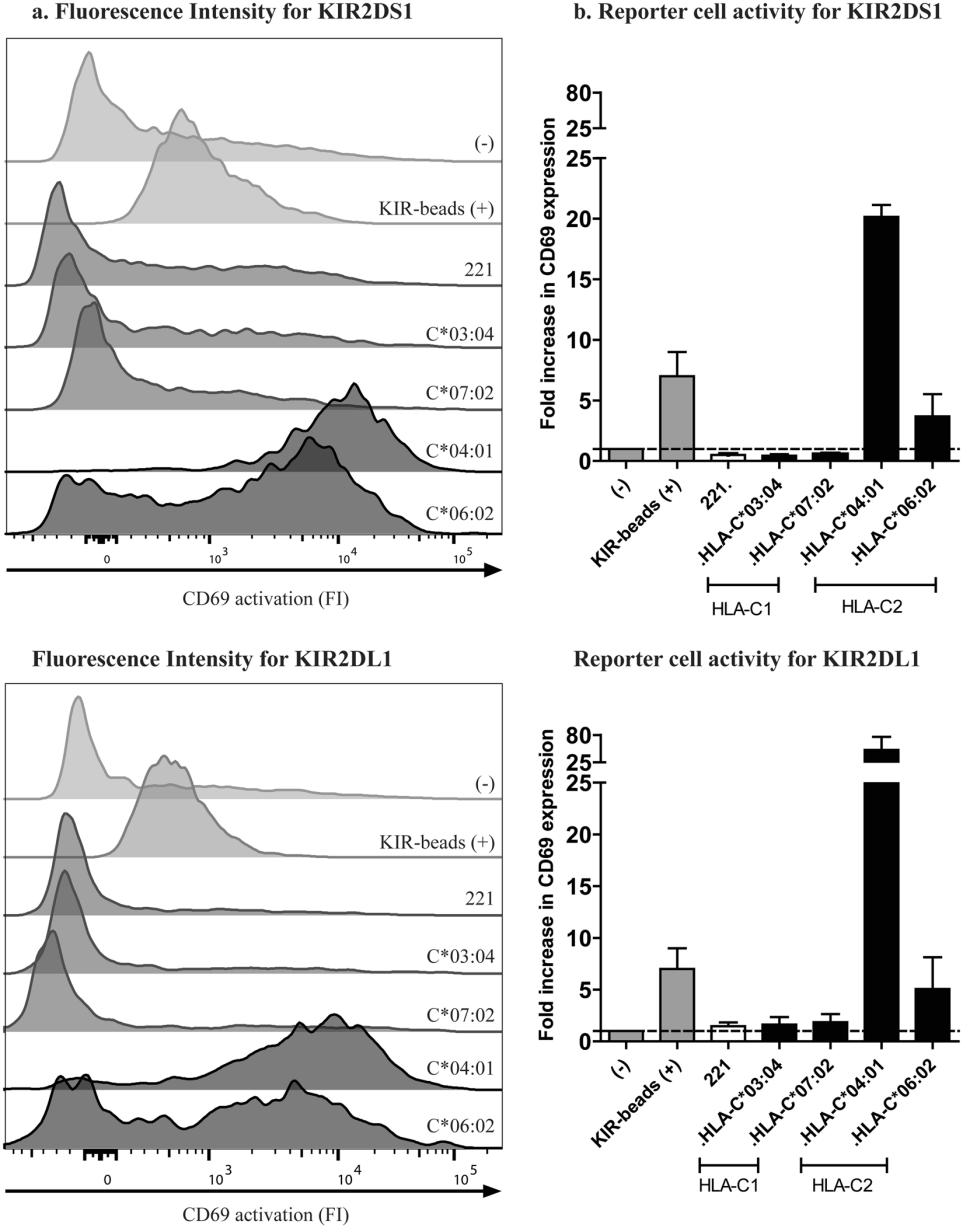
Binding of KIR2DS1 and KIR2DL1 reporter cell lines to HLA-C cell lines. KIR2DS1ζ^+^ or KIR2DL1ζ^+^ Jurkat cells were incubated with 721.221 cell lines transduced with HLA-C*03:02 or HLA-C*07:02 (HLA-C1), HLA-C*04:01 or HLA-C*06:02 (HLA-C2). KIR2DS1ζ^+^ or KIR2DL1ζ^+^ Jurkat cells alone were used as negative control (−) and KIR2DS1ζ^+^ or KIR2DL1ζ^+^ Jurkat cells pulsed with KIR2D-coupled beads were used as positive control (+). (**a**) Representative flow cytometry histogram showing Fluorescence Intensity (FI) for CD69 activation for KIR2DS1ζ^+^ and KIR2DL1ζ^+^ Jurkat cells. (**b**) Bar graph showing fold increase in CD69 expression (MdFI of the sample divided by the MdFI of KIR2DS1ζ^+^ or KIR2DL1ζ^+^ Jurkat cells stained in the absence of target cells). Each bar represents median +/− interquartile range of 3 independent experiments.

To determine the functional consequences of the above observed binding between KIR2D and HLA-C molecules, KIR2DS1ζ^+^ and KIR2DL1ζ^+^ -expressing Jurkat reporter cells were incubated with 721.221 cells stably transduced with specific HLA-C1 ligands (HLA-C*03:04, HLA-C*07:02) or HLA-C2 ligands (HLA-C*04:01, HLA-C*06:02) (Fig. 2a and b). HLA-devoid 721.221 cells were used as negative controls. Ligand engagement of KIR2DS1ζ^+^- or KIR2DL1ζ^+^-expressing Jurkat cells resulted in an activating signal through CD3ζ that triggered CD69 expression. Beads coupled with KIR2DL1/S1 (HPMA4) antibodies were used as a positive control. KIR2DS1ζ^+^ Jurkat cells were significantly activated by 721.221. cells expressing HLA-C*04:01 or HLA-C*06:01, while 721.221- cells expressing HLA-C*03:04 and HLA-C*07:02 did not activate KIR2DS1ζ^+^ Jurkat cells. KIR2DL1ζ^+^ Jurkat cells were activated by 721.221- cells expressing HLA-C*04:01 and HLA-C*06:02 to a higher degree, while 721.221- cells expressing HLA-C*03:04 and HLA-C*07:02 only weakly activated KIR2DL1ζ^+^ Jurkat cells. This results confirmed the HLA-I/KIR binding screening data, with KIR2DS1ζ^+^ and KIR2DL1ζ^+^ Jurkat cells being both activated by 721.221- cell lines expressing HLA-C2 ligands, and KIR2DL1ζ^+^ Jurkat cells being more strongly activated than KIR2DS1ζ^+^ Jurkat cells.

### KIR2DS1 and KIR2DL1 binding to HLA-C2 molecules is peptide-dependent

Several studies have revealed that peptides presented by HLA class I molecules can influence KIR binding^19–21, 25^. We therefore investigated whether specific peptides presented by the HLA-C2 allele HLA-C*06:02 can impact KIR2DS1- as well as KIR2DL1-binding. To prevent 721.221-HLA-C*06:02 cells from presenting self-peptides onto HLA-C*06:02, 721.221-HLA-C*06:02 cells with a knock-out for TAP1 were produced. This allowed for the controlled identification of externally added peptides that bind to and stabilize HLA-C*06:02 expression on the cell surface. HLA stabilization was quantified by measuring HLA expression on 721.221.TAP1KO-HLA-C*06:02 cells using flow cytometry.

**Figure 3:**
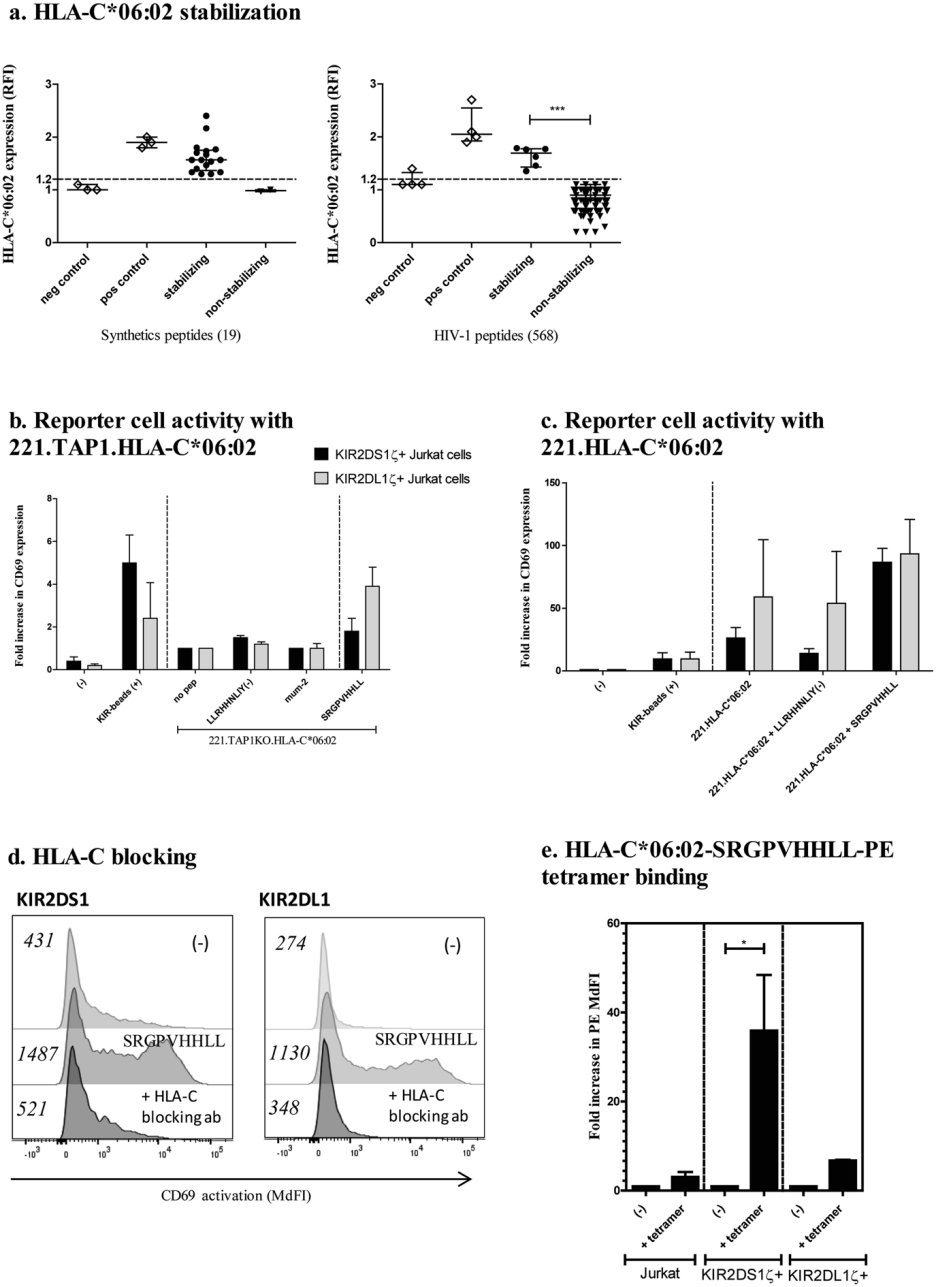
Impact of peptide presented by HLA-C*06:02 on KIR2DS1 and KIR2DL1 binding. (**a**) Quantification of HLA-C*06:02 stabilization of 721.221-TAP1KO-C*06:02 pulsed with 19 different synthetic peptides and 568 HIV-1 clade B peptides. HLA-C*06:02 surface levels were determined by flow cytometry using an anti-pan-HLA class I antibody (clone W6/32). Peptides were added at a saturating concentration of 200 μM. Relative fluorescence intensity (RFI) was calculated as the pan-HLA MdFI of the sample divided by the pan-HLA MdFI of 721.221-TAP1KO-C*06:02 cells stained in the absence of peptide. Binding peptides were determined as (Mean + S.D.)_MFI sample_ > (Mean + 2*S.D.)_MFI LIY_. The dotted line represents the cut-off set to determine the peptides binding to HLA-C*06:02. The screening was performed one time and the selected peptides were tested in three independent experiments. (**b**) Bar graphs showing fold increase in CD69 expression for KIR2DS1ζ^+^ (black bar) and KIR2DL1ζ^+^ (grey bar) Jurkat cells when co-incubated with 721.221-TAP1KO-C*06:02 pulsed with different peptides (MdFI of the sample divided by the MdFI of KIR2DS1ζ+ or KIR2DL1ζ+ Jurkat cells co-incubated with 721.221-TAP1KO-C*06:02 in the absence of peptide). (**c**) Bar graphs showing fold increase in CD69 expression for KIR2DS1ζ^+^ (black bar) and KIR2DL1ζ^+^ (grey bar) Jurkat cells when co-incubated with 721.221-C*06:02 pulsed with different peptides (MdFI of the sample divided by the MdFI of KIR2DS1ζ^+^ or KIR2DL1ζ^+^ Jurkat cells alone). (**d**) HLA-C blocking antibody (6A4) abrogated CD69 activation of the KIR2DS1ζ^+^ and KIR2DL1ζ^+^ Jurkat cells after pre-incubation with the indicated target cells and subsequent incubation with the KIR2DS1ζ^+^ or KIR2DL1ζ^+^ Jurkat cells. The experiments were repeated three times independently and a representative example is shown with the mean of the CD69 FI. (**e**) HLA-C*06:02- SRGPVHHLL -PE tetramer staining of Jurkat- β2mKO, KIR2DS1ζ^+^ or KIR2DL1ζ^+^ Jurkat cells. Results are shown as fold increase in PE MdFI (MdFI of the reporter cell line stained with the tetramer divided by the MdFI of the reporter cell line alone). For each bar graphs, the results are shown as median of three independent experiments +/− interquartile range.

We first tested 19 synthetic peptides that had been previously described to bind to HLA-C*06:02^26^. Furthermore, 568 overlapping peptides spanning the entire HIV-1 clade B sequence peptides (346 18aa-long peptides covering the entire HIV-1 consensus sequence and 222 decametric peptides overlapping by 9 amino acid and covering p24 GAG) were assessed for their potential to stabilize HLA-C*06:02. Peptides inducing a robust increase of HLA-C*06:02-expression (MdFI higher than 2 S.D. above the mean of non-stabilizing control peptide (LLRHHNLIY)) were defined as HLA-C*06:02-binding peptides, resulting in the identification of 20 peptides presented by HLA-C*06:02 (Fig. 3a). The peptides included 14 of the 19 previously described synthetic HLA-C*06:02-binding peptides and 6 novel HIV-1-derived peptides. Most of the peptides stabilizing HLA-C*06:02 showed the same binding motif consisting of a phenylalanine in position 1 of the peptide sequence (15/20), an arginine in position 2 of the peptide sequence (15/20) and an aliphatic amino acid (valine, leucine or isoleucine) in position 9 of the peptide sequence (16/20), which is in agreement with the previously defined binding motif for HLA-C*06:02^26–28^ (see Supplementary Table 1).

The 20 selected peptides that stabilized HLA-C*06:02 expression were subsequently tested for their ability to engage KIR2DS1- and KIR2DL1 by co-incubating KIR2DS1ζ^+^ and KIR2DL1ζ^+^ Jurkat reporter cells with 721.221-TAP1KO- HLA-C*06:02 cells loaded with the respective peptides (Fig. 3b). Only one out of the 20 tested peptides, SRGPVHHLL (HLA-Cw6-SV9), showed a significant increase of reporter cell activity for both KIR2DS1ζ^+^ and KIR2DL1ζ^+^ Jurkat cell lines. None of the 6 identified HLA-C*06:02-binding HIV-1 peptides induced any activation of Jurkat reporter cells lines. The SRGPVHHLL peptide furthermore increased the reporter cell activity of both KIR2DS1ζ^+^ and KIR2DL1ζ^+^ Jurkat cells in response to TAP-competent 721.221.HLA-C*06:02 cells (Fig. 3c). Blocking experiments (Fig. 3d) were performed by pre-incubation of 721.221.TAP1KO-HLA-C*06:02 cells pulsed with the peptide SRGPVHHLL with an HLA-C blocking antibody (clone 6A4, IgM) before incubation with KIR2DS1ζ^+^ and KIR2DL1ζ^+^ Jurkat cells and resulted in the abrogation of reporter cell activity, confirming that KIR2DS1ζ^+^ and KIR2DL1ζ^+^ Jurkat cells activation was HLA/peptide-dependent.

To further confirm our findings, HLA-C*06:02 tetramers folded with the peptide SRGPVHHLL and conjugated to the fluorescent molecule PE (referred as HLA-C*06:02-SRGPVHHLL-PE) were used to stain the KIR2DS1ζ^+^ and KIR2DL1ζ^+^ Jurkat cells (Fig. 3e). A strong increase of PE MdFI signal was observed when the KIR2DS1ζ^+^ and KIR2DL1ζ^+^ Jurkat cells were stained with the HLA-C*06:02-SRGPVHHLL-PE tetramer. Taken together, out of the 587 peptides tested, we identified one synthetic peptide, SRGPVHHLL, which stabilized HLA-C*06:02-expression and induced strong and functionally relevant binding of KIR2DS1 and KIR2DL1.

### Amino acid variations within the HLA-C*06:02-restricted SRGPVHHLL peptide impact KIR2DS1 binding

**Figure 4:**
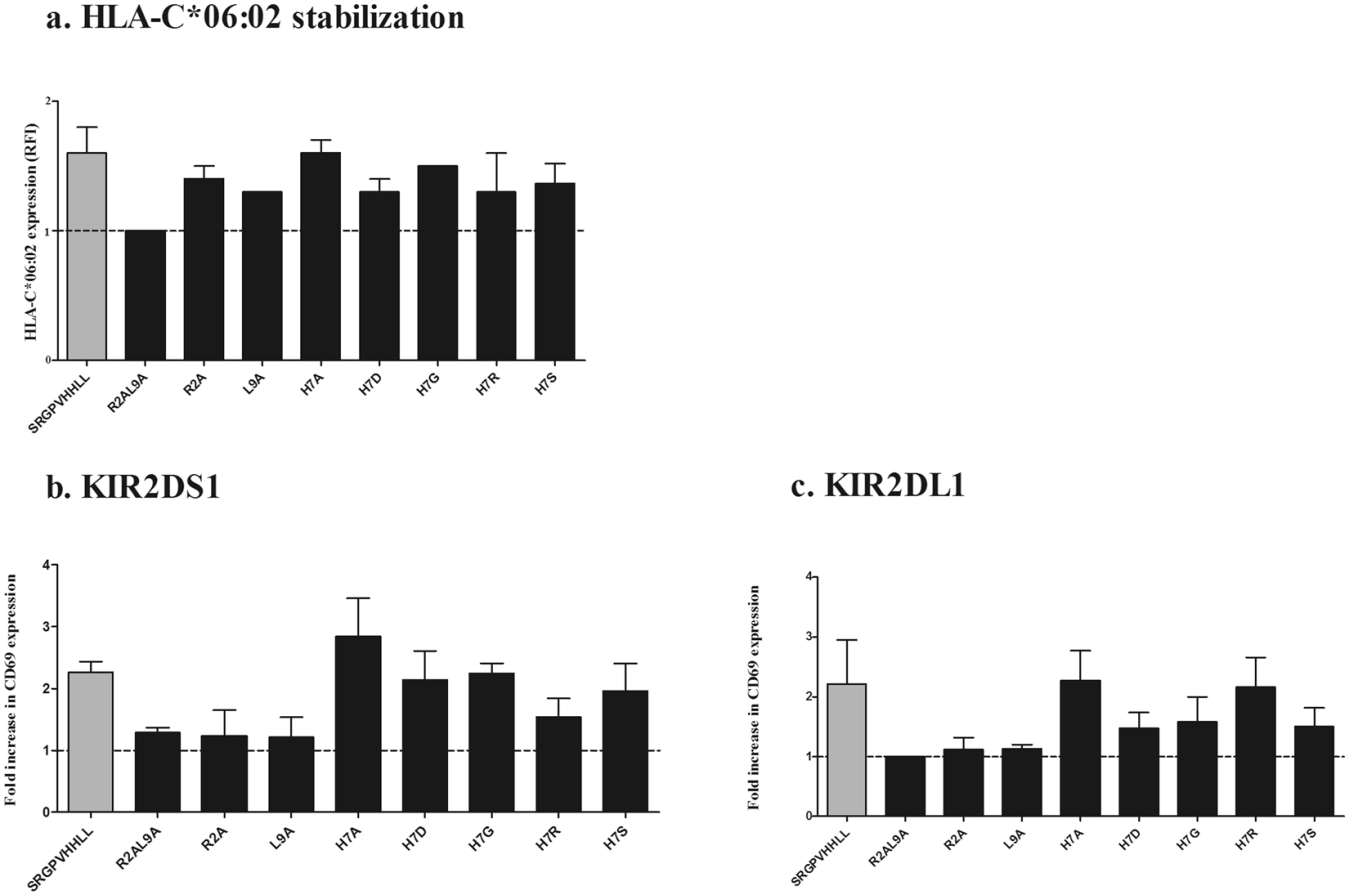
Impact of amino acid modification of the peptide SRGPVHHLL presented by HLA-C*06:02 on KIR2DS1 and KIR2DL1 binding. (**a**) Quantification of HLA-C*06:02 stabilization of 721.221.TAP1KO- HLA-C*06:02 pulsed with the indicated peptides. Peptides were added at a saturating concentration of 200 μM. Of note, the median +/−S.D. of R2AL9A, L9A and H7G were respectively 1 ± 0, 1.3 ± 0.057 and 1.5 ± 0.057 (**b**) and (**c**) Bar graph showing fold increase in CD69 for KIR2DS1ζ^+^ or KIR2DL1ζ^+^ Jurkat cells when co-incubated with 721.221-TAP1KO-C*06:02 pulsed with different peptides (MdFI of the sample divided by the MdFI of KIR2DS1ζ^+^ or KIR2DL1ζ^+^ Jurkat cells co-incubated with 721.221-TAP1KO-C*06:02 in the absence of peptide). Each bar represents median +/− interquartile range of 3 independent experiments.

To determine the peptide residue important for modulating KIR2DS1- and KIR2DL1-binding, various amino acid substitutions were introduced in the SRGPVHHLL peptide sequence. A panel of 8 peptides (see Supplementary Table 2) was synthetized with amino acid substitutions at P2 or P9, identified previously as anchor residues for HLA-C*06:02-binding^26–28^, or P7, a position described as important for KIR recognition^19–21^. The amino acid substitutions were selected to cover the principal categories of amino acids according to their side chain (polar, apolar, neutral, acidic, basic). Peptides were first tested for HLA-C*06:02 stabilization and 7 out of the 8 peptides were identified to stabilize HLA-C*06:02-expression, demonstrating that most single amino acid substitution did not affect HLA-C*06:02-binding (Fig. 4a). The peptide R_2_A/L_9_A did not show any stabilization of HLA-C*06:02, suggesting that amino acid substitutions in both anchor position (P2 and P9) abrogated HLA-C*06:02-binding. Subsequently, the peptide variants were tested for their ability to modify KIR2DS1ζ^+^ and KIR2DL1ζ^+^ binding when presented by HLA-C*06:02 (Fig. 4b and c). The peptide R_2_A/L_9_A, which did not stabilize HLA-C*06:02, was used as an additional negative control. The peptides R_2_A and L_9_A, which stabilized HLA-C*06:02-expression, decreased both KIR2DS1ζ^+^ and KIR2DL1ζ^+^ Jurkat cell activity to the same level as the negative control peptide (LLRHHNLIY), showing that single substitution in P2 or P9 abrogated KIR2DS1 as well as KIR2DL1 binding. The peptides with the sequence changes H_7_A, H_7_G and H_7_S did not affect KIR2DS1ζ^+^ and KIR2DL1ζ^+^ Jurkat cell reporter activity compared to wild type peptide. Finally, the peptide with the H_7_R substitution slightly decreased the reporter cell activity of KIR2DS1ζ^+^ Jurkat cells, but not the activity of KIR2DL1ζ^+^ Jurkat cells, while the peptides with the H_7_D and H_7_S substitutions slightly decreased the reporter cell activity of KIR2DL1ζ^+^ Jurkat cells, but not of KIR2DS1ζ^+^ Jurkat cells. In summary, we demonstrated that binding of KIR2DS1 and KIR2DL1 to the SRGPVHHLL peptide presented by HLA-C*06:02 was modulated by the peptide sequence.

### SRGPVHHLL presented by HLA-C*06:02 triggers degranulation of primary KIR2DS1+NK cell clones

**Figure 5:**
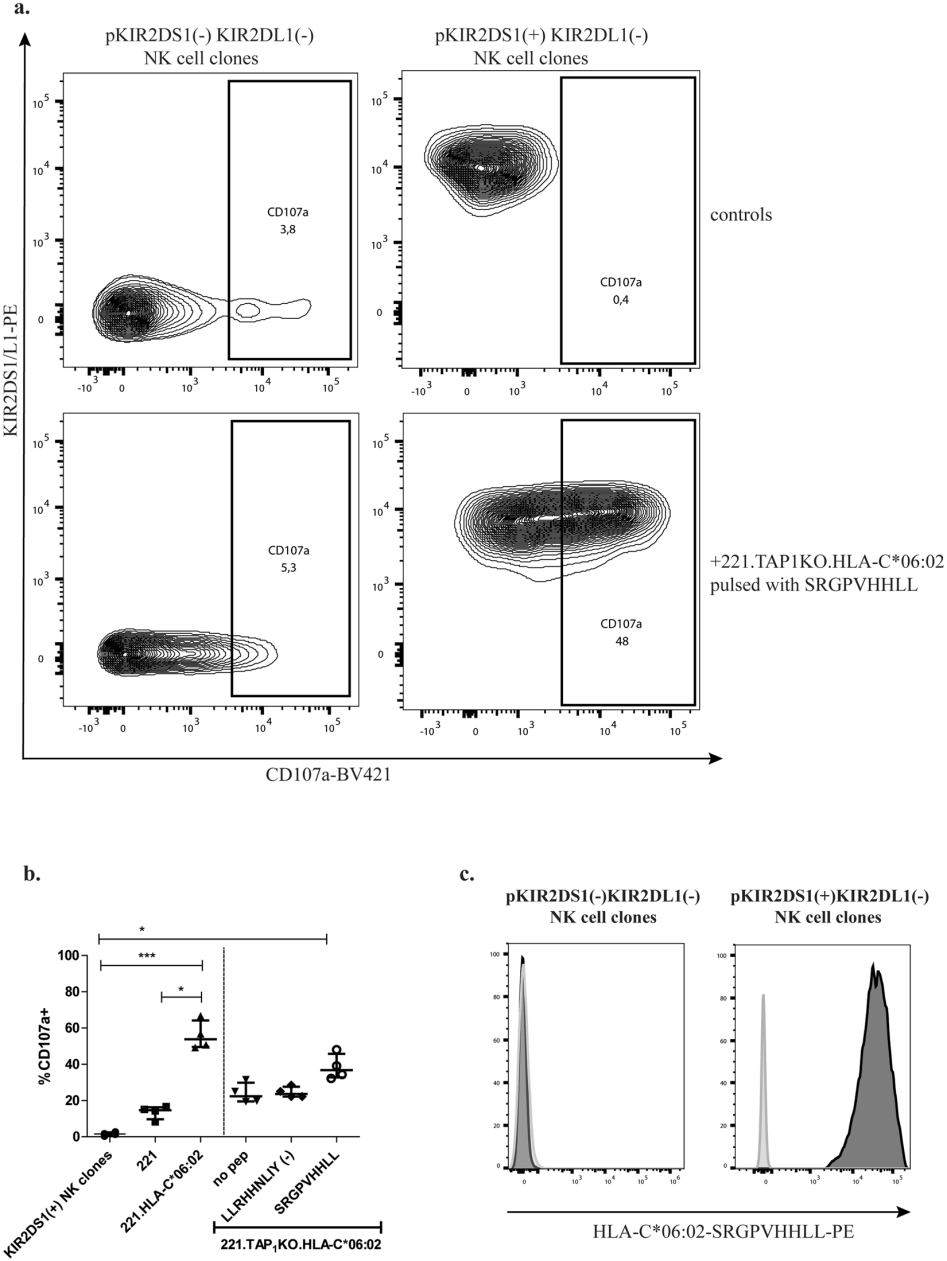
Functional impact of peptide SRGPVHHLL on primary KIR2DS1(+) KIR2DL1(−) and KIR2DS1(−)KIR2DL1(−) NK cell clones. (**a**) Representative example of CD107a production by primary KIR2DS1(+) KIR2DL1(−) and KIR2DS1(−) KIR2DL1(−) NK cell clones cultured alone or in presence of 721.221.TAP1KO- HLA-C*06:02 pulsed with 100 µM of the peptide SRGPVHHLL (**b**) Paired comparison of the percentage of CD107a NK cells after 5hrs stimulation of clonal KIR2DS1(+) KIR2DL1(−) NK cells with target cells. The experiments were repeated 4 times independently and showed are the median +/− interquartile range. (**c**) Representative example of HLA-C*06:02- SRGPVHHLL -PE tetramer staining of primary KIR2DS1(+) KIR2DL1(−) or KIR2DS1(−) KIR2DL1(−) NK cell clones. Primary KIR2DS1(+) KIR2DL1(−) NK cell clones incubated without the tetramer are represented in grey whereas the cells stained with the tetramer are represented in black.

The functional consequences of peptide-dependent engagement of activating KIRs remains insufficiently understood. The effects of the SRGPVHHLL peptide presented by HLA-C*06:02 on KIR2DS1 binding was therefore investigated by performing NK cell degranulation assays using primary KIR2DS1(+) KIR2DL1(−) and KIR2DS1(−) KIR2DL1(−) NK cell clones derived from KIR2DS1+ HLA-C1/C1 individuals (see Supplementary Fig. 1 for gating strategy and characterization of NK cells clones). As displayed in Fig. 5a and b, 721.221-TAP1KO- HLA-C*06:02 cells pulsed with the SRGPVHHLL peptide induced strong degranulation of KIR2DS1(+) KIR2DL1(−) NK cell clones, as measured by the percentage of CD107a(+) NK cells, compared to 721.221.TAP1KO-HLA-C*06:02 cell alone or pulsed with the control peptide LLRHHNLIY. In contrast, KIR2DS1/KIR2DL1 double-negative NK cell clones were not activated by the peptide SRGPVHHLL. Moreover, HLA-C*06:02-tetramers refolded with the SRGPVHHLL peptide stained KIR2DS1(+) KIR2DL1(−) NK cell clones, while no binding to KIR2DS1(−) KIR2DL1(−) NK cell clones was observed (Fig. 5c). Of note, as previous studies demonstrated that the co-expression of the inhibitor receptors KIR2DL2/KIR2DL3, KIR3DL1 and NKG2A can affect the outcome of KIR2DS1(+) NK cell degranulation^29^, we phenotyped all NK cell clones for these receptors. The presence or absence of KIR2DL2/KIR2DL3, KIR3DL1 and NKG2A on these clones did not affect the results (data not shown). All together, these data demonstrate that the HLA-C*06:02 peptide SRGPVHHLL enabled KIR2DS1-binding and resulted in the activation of KIR2DS1(+), but not KIR2DS1(−) NK cell clones derived from the same individual.

## Discussion

The factors that determine the engagement of the activating NK cell receptor KIR2DS1 remain incompletely understood despite a growing number of genetic studies showing associations between KIR2DS1 and the outcome of various human diseases. In this study, we investigated the HLA class I molecules and HLA class I-presented peptides enabling KIR2DS1-binding and their influence on KIR2DS1(+) NK cell function. We demonstrate that KIR2DS1-binding is narrowly restricted to HLA-C2 molecules whereas KIR2DL1 showed a broader binding specificity for HLA-C2 but also for some HLA-C1 and HLA-B molecules. HLA-C2-presented peptides modulated both KIR2DS1- and KIR2DL1-binding to HLA-C2, and we identified one HLA-C*06:02-presented peptide (SRGPVHHLL) that strongly engaged KIR2DS1 and also KIR2DL1 binding. This synthetic peptide, predicted to bind HLA-C*06:02, did not correspond to any known human or viral sequence, and strongly activated primary KIR2DS1(+) NK cell clones in a peptide sequence-specific manner. Taken together, these data demonstrate peptide-dependent activation of the activating NK cell receptor KIR2DS1 and its inhibitory counterpart KIR2DL1.

A broad HLA class I-binding screen performed in this study demonstrated that KIR2DL1 and KIR2DS1 exhibited very similar binding specificities to HLA-C2 molecules, consistent with previous studies^16, 17, 22^. However KIR2DL1 showed a higher binding affinity for these HLA-C2 ligands, and a broader HLA class I binding specificity than KIR2DS1, as it also bound weakly to eight HLA-C1 complexes (HLA-C*01:02; *03:02; *03:03; *03:04; *07:02; *08:01; *12:03; *16:01) as well as two HLA-B complexes (HLA-B*46:01 and HLA-B*73:01). Given the opposite functions of the activating KIR2DS1 receptor and the inhibitory KIR2DL1 receptor, a very restricted set of ligands for the activating NK cell receptor KIR2DS1 might limit the risks of auto-immune reaction. Furthermore, both KIR2DS1 and KIR2DL1 shared the same pattern of peptide-specific recognition, which is consistent with previous studies^17, 22, 23, 48^ as they bound to the same HLA-C*06:02-presented peptide SRGPVHHLL. The functional assays performed using KIR2DS1ζ^+^ and KIR2DL1ζ^+^ Jurkat reporter cells also showed that KIR2DL1 had a stronger binding affinity than KIR2DS1, consistent with previous studies showing that inhibitory receptors have a stronger binding affinity than their corresponding activating NK cell receptor^17, 30^ for the same ligand. Of note, KIR2DL1 and KIR2DS1 are not always present at the surface of the same NK cell as KIR receptors are expressed stochastically, and it has been shown that around 10% of circulating NK cells expressed KIR2DS1 in absence of KIR2DL1^31^. Our data suggest that this subset of KIR2DS1(+) KIR2DL1(−) NK cells is able to mediate an effective effector function in response to their ligands. Notably, the tetramer staining performed using HLA-C*06:02 molecules refolded with the peptide SRGPVHHLL showed higher binding affinity for KIR2DS1 compared to KIR2DL1. This result might be linked to the observation that KIR2DS1 can assemble in larger clusters than KIR2DL1 at the surface of cells^32^, potentially favoring a stronger binding of HLA class I tetramers. Overall, our data showed that the NK cell receptors KIR2DS1 and KIR2DL1 shared the same ligand specificity for HLA-C2-presented peptides, but differed in their binding affinity.

Several studies using crystal structures of KIR2DL1^21^, KIR2DL2^19^, KIR2DL3^20^ and KIR2DS2^25^ have described that the KIR binding interactions with HLA class I presented epitopes are centered to the COOH-terminal end of the peptide which corresponds to peptide residues P7-P8^33^. In contrast, the specific recognition of HLA-presented peptide by TCR depends on the sequence of the entire peptide and is centered at the P4-P6 positions^34^. Our results showed that peptide residue P7 of the HLA-C*06:02 presented peptide SRGPVHHLL can modulate KIR2DS1 and KIR2DL1 binding, as previously described^22^, but that amino acid changes in residues P2 and P9 can also impact the binding of KIR2DS1 and KIR2DL1. The residues P2 and P9 are defined as anchor residues for HLA-C*06:02 binding^26–28^. Modifications of these residues may induce conformational changes of the peptide SRGPVHHLL presented by HLA-C*06:02, which can abrogate the binding to KIR2DS1 and KIR2DL1. The resolution of the crystal structure of KIR2DS1 in conjunction with HLA-C molecules presenting specific peptides will help clarifying these interactions. In summary, our results suggest that the HLA-C*06:02-presented peptide SRGPVHHLL can modulate KIR2DS1 and KIR2DL1 in a peptide sequence-specific manner.

Very few studies have investigated the ability of HLA class I-presented peptides to facilitate the binding of activating KIRs. Here we demonstrate that KIR2DS1-activation can be modulated by HLA-C2-presented peptide﻿s; however, despite screening over 500 virus-derived peptides, we did not identify any viral peptides binding to KIR2DS1. The peptide SRGPVHHLL identified to enable strong KIR2DS1-binding to HLA-C*06:02 was derived from a synthetic peptide library predicted to bind to HLA-C*06:02^26^, and does not match any known viral or human epitope. Several HIV-1-derived peptides presented by their respective HLA class I molecules have been demonstrated to modulate the binding of inhibitory KIRs, including KIR2DL2^35, 36^, KIR2DL3^37^ and KIR3DL1^38, 39^. Thus, it is remarkable that none of these peptides was able to engage the binding of KIR2DS1. Studies have indicated that viruses are able to select for sequence variants that enhanced the binding of inhibitory KIRs and thus inhibit the effector function of KIR+ NK cells, suggesting that viruses are able to escape NK cell-mediated immune pressure^38–40^. It might therefore be possible that HIV-1 has eliminated sequences that do allow binding to the activating KIR2DS1 receptor when presented by the HLA-C*06:02 molecule. To our knowledge, the only viral peptide presented by HLA class I and binding to an activating KIR that has been identified is a vaccinia virus-derived peptide in complex with HLA-A*11 binding to KIR2DS2^25^. Additionally, direct binding of primary activating KIR2DS1+ NK cells to virally infected cells has only been shown for EBV-transformed 221.HLA-C*04:01^17^, but no specific viral peptide was identified. Taken together, viruses might thus have evolved specific mechanisms to avoid recognition of viral peptides by activating NK cell receptors.

The role of activating KIRs has been extensively studied in the context of allogeneic stem cell transplantation to treat leukemia, especially in the setting of KIR ligands-mismatched donor/recipient pairs^41–44^. In particular, donor-derived KIR2DS1+ NK cells were shown to efficiently kill HLA-C2+ leukemia blasts, which indicate that activating KIRs can have a role in mediating anti-leukemic or anti-cancer effects^29^. The HLA-C*06:02 presented peptide SRGPVHHLL identified in this study mediated strong activation of primary NK cell clones, suggesting that this peptide could be potentially used to label specific tumors in order to enhance anti-tumor cytotoxic activity by KIR2DS1(+) NK cells. The use of the synthetic SRGPVHHLL peptide as an enhancer for KIR2DS1(+) NK cell-mediated cytotoxicity might therefore provide a new perspective for NK cell immunotherapy.

## Methods

### Cell lines and PBMC

The HLA-class-I deficient 721.221 B cell line was used to produce the cell lines 721.221-HLA-C*06:02, -HLA-C*04:01, -HLA-C*03:04 and -HLA-C*07:02 using methods already described^46^. The 721.221.TAP1KO-HLA-C*06:02 cell line was generated by knocking out the TAP1 gene using CRISPR/CAS9 technology. Briefly, 721.221-HLA-C*06:02 were transduced with lentiCas9-Blast (Addgene plasmid # 52962) and selected in 5 μg/mL blasticidin S (Sigma-Aldrich). 721.221-HLA-C*06:02.Cas9 cells were transduced with TAP1 gRNA lentivirus (Addgene plasmid # 52962)), selected in 200 μg/ml Neomycin (Sigma-Aldrich) and sorted for loss of HLA expression. LentiCas9-Blast and TAP1 gRNA lentiviruses were kindly provided by Feng Zhang^45^. The Jurkat cell lines (clone E6, ATCC) were used to generate Jurkat-β2 mKO-KIR2DS1 cells (referred as KIR2DS1ζ^+^ Jurkat cells) and Jurkat-β2mKO-KIR2DL1 cells (referred as KIR2DL1ζ^+^ Jurkat cells). The Jurkat cell lines were produced via knocking out of the β2m as already described^46^. KIRζ chimeric constructs with the extracellular domain of KIR2DS1*002 or KIR2DL1*001 (Gene Art) were cloned into a lentiviral transfer vector containing an SFFV promoter and IRES-driven puromycin resistance. Lentiviral supernatant produced by three-plasmid transfection (psPAX2, VSV-G and transfer vector) of 293T cells via lipofectamine^37^ was obtained to transduce Jurkat- β2mKO cells. 72 h post-transduction, the cell lines Jurkat- β2mKO.KIR2DS1 and Jurkat- β2mKO.KIR2DL1 were selected in 2 μg/ml Puromycin (Sigma-Aldrich) and sorted for high expression of KIR. Human primary blood mononuclear cells (PBMC) were isolated from healthy donors recruited at the University medical Centre Hamburg-Eppendorf, using density centrifugation. Each participant gave informed consent prior to enrollment. All cell lines were cultivated at 37 °C under 5% CO2 in RPMI medium 1640 (Sigma-Aldrich) supplemented with 10% heat-inactivated fetal bovine serum (Sigma-Aldrich), 2,500 U/ml penicillin, 2,500 μg/ml streptomycin, and 100 mML-glutamine (Cellgro) (referred as RP10). All methods were performed in accordance with the relevant guidelines and regulations and approved by the ethical commission of the Ärztekammer Hamburg.

### Antibodies

The following antibodies were used for cellular assays: anti-HLA-ABC-APC (clone W6/32, eBioscience), anti-CD3-BU737 (clone UCHT1, BD Bioscience), anti-CD3-PerCyP.5.5 (clone UCHT1, BioLegend), anti-CD19-BV510 (clone HIB19, BioLegend), anti-CD56-BU395 (clone NCAM16.2, BD Bioscience), anti-CD16-BV785 (clone 3G8, BioLegend), anti-KIR2DL1/S1/L3/S3/L5/S5-PE (clone HP-MA4, eBioscience), anti-KIR2DL1/S1-PE (clone 11PB6, Miltenyi), anti-KIR2DL1-FITC (clone REA284, Miltenyi), anti-KIR2DL3-APC (clone DX27, Miltenyi), anti-KIR3DL1/S1-BV421 (clone DX9, BioLegend), anti-NKG2A-PE-Cy7 (clone Z199, Beckman), anti-NKG2D-APC (clone 1D11, BioLegend), goat anti-human IgG(Fc) F(ab´)-PE (Life Technologies), anti-CD69-BV421 (clone FN50, BioLegend), anti-CD107a-BV421 (clone M4A3, BioLegend). For blocking assays we pre-incubated the samples with anti-HLA-C antibodies (clone 6A4, IgM, kindly provided by Prof. L. Moretta, Istituto Giannina, Genova, Italia) diluted 1/20 for 20 min at 37 °C.

### KIR-Fc binding assay to HLA-I coated beads

Screening of classical HLA-I coated beads was performed using LABScreen Single Antigen HLA Class I - Combi (One Lambda) according to the manufacturer’s instructions. 40 μl of indicated concentration of KIR2DS1-Fcs (allele KIR2DS1*002) and KIR2DL1-Fcs (allele KIR2DL1*001) (R&D system) diluted in PBS were added to a 96-well plate and incubated with a mixture of 97 classical anti-human HLA-A, B, C beads for 30 mn at room temperature. The plate was washed and the samples were incubated with goat- anti-human IgG-PE secondary antibody for 30 mn at room temperature. Finally, KIR-Fc binding to the beads was analyzed on a Bio-Plex 200 (Bio-Rad Laboratories). Results for KIR-Fc binding to HLA-I coated beads were presented as Median Fluorescence Intensity (MdFI) of the KIR-Fc binding.

### Peptide binding assay for HLA-C stabilization

HLA Class I -stabilization assays were performed as previously described^46^. 721.221.TAP1KO- HLA-C*06:02 were incubated 24 hrs in serum-free RPMI 1640 to remove any remaining peptides from the RP10 medium. Cells were washed twice and pulsed with 200 µM of the indicated peptides for 20 hrs at 37 °C in serum-free medium (referred as “R0”). The staining was performed using the Zombie Aqua fixable viability kit (BioLegend) following the manufacturer´s instructions and anti HLA-ABC-APC to quantify HLA class I expression. After fixation in 4% paraformaldehyde, samples were analyzed by flow cytometry (BD LSR Fortessa). The 721.221-TAP1KO- HLA-C*06:02 incubated with no peptide as well as the HCV peptide, LLRHHNLIY were used as negative control. As positive controls, 721.221-HLA-C*06:02 cell line, which stably expresses HLA-C*06:02, and the MUM-2 derived peptide, FRSGLDSYV, already described as HLA-C*06:02 binder^26^ were used.

### KIR-Jurkat reporter cell assay

KIRζ chimeric constructs for the generation of the KIR2DL1ζ^+^ Jurkat reporter cells consisted of the extracellular and transmembrane domains of KIR2DL1*001 linked to the cytoplasmic tail of CD3ζ. KIR2DS1ζ^+^ Jurkat reporter cells contained the extracellular domain of KIR2DS1*002, the transmembrane domains of KIR2DL1*001 linked to the cytoplasmic tail of CD3ζ. A sequence coding for the Zs Green protein was added to the KIR2DS1 chimeric construct to allow the differentiation between the KIR2DL1 and KIR2DS1 reporter cell line by GFP signal. Ligand engagement of KIR2DL1 or KIR2DS1 resulted in an activating signal that triggered CD69 expression. Measurement of KIR binding was assessed as Median Fluorescence Intensity (MdFI) of CD69. Reporter cells line were cultured overnight in RP20 (RPMI supplemented with 20% FBS) at 2.5*10^5^ cells/ml to reduce background activation. KIR2DS1ζ^+^ Jurkats cells or KIR2DL1ζ^+^ Jurkats cells were co-incubated with target cells pulsed with peptides at a ratio effector/target 1/10 for 3 hrs at 37 °C under 5% CO2 in RP20. Cells were washed and stained for anti-CD3-BUV737, live/dead-BV510, anti-KIR2DL1/S1/L3/S3/L5/S5-PE (HPMA4) and anti-CD69-BV421 20 mn at room temperature in the dark. After fixation in 4% paraformaldehyde, the samples were analyzed by flow cytometry (BD LSR Fortessa). KIR2DS1ζ^+^ or KIR2DL1ζ^+^ -Jurkat cells were used as negative control and KIR2DS1ζ^+^ or KIR2DL1ζ^+^ -Jurkat cells pulsed with 10 μl of HPMA4-conjugated beads (Dynabeads M-450 Tosylactivated, Invitrogen, conjugated following the manufacturer’s instructions) were used as positive control.

### Generation of clonal NK cells

Primary NK cell clones were generated from PBMCs from healthy donors with a KIR2DS1+ and HLA-C1 homozygous genotype using methods described^46^. We isolated NK cells using EasySep™ Human NK Cell Enrichment Kit (StemCell) following manufacturers protocol. NK cells were cultivated overnight in RP10 supplemented with 500 U/ml IL2. Next day, they were resuspended in cloning medium consisting of RPMI supplemented with 10% fetal bovine serum (Sigma-Aldrich), 5% human serum (Sigma-Aldrich), 2 mM L-glutamine (Gibco), 1X MEM-NEAA (Gibco), 1X sodium pyruvate (Gibco), 100 μg/mL Primocin™ (Invivogen), 500 U/mL IL-2 (Sigma Aldrich), with the addition of the following four cytokines: 5 ng/mL IL-15 (Peprotech), 10 ng/mL IL-12 (Peprotech), 40 ng/mL IL-18 (Peprotech), and 20 ng/mL IL-21 (Peprotech). NK cells were stained for anti-CD3-BUV737, anti-CD19-BV510, anti-CD56-BUV395, anti-CD16-BV785, anti-KIR2DL1-S1-PE (EB6) and anti-KIR2DL1-FITC (REA284) and sorted for single cells in 96 well plates using FACS ARIA Fusion to produce 2 different subsets of NK cell clones: KIR2DS1(+) KIR2DL1(−) and KIR2DS1(−) KIR2DL1(−). After sorting, 100 µl of feeders cells consisting of irradiated K562 cells expressing mbIL-15 and CD137L (kind gift from Dario Campana^47^) and irradiated allogeneic PBMC homozygous for HLA-C1 (ratio K562/PBMC 1/10) were added to the wells. After 10 days, 200 μL of NK cloning medium was added to the growth pellets. After 3 days, NK cell clones were harvested and transferred in 24 well plates with NK cloning medium and 500 U/ml IL2. After 3 supplementary days, NK cell clones were phenotyped and used for assays. To ensure proper functionality, NK cell clones were also phenotyped for NKG2A, KIR2DL2/3 and KIR3DS1.

### NK cell degranulation assay

The day before the experiment, KIR2DS1(+) KIR2DL1(−) and KIR2DS1(−) KIR2DL1(−) NK cell clones were cultivated in NK cloning medium without IL-2 overnight to avoid background activation levels. The NK cell clones were co-incubated with target cells lines, previously pulsed with 100 µM peptide for 20 hrs, in the presence of 2 µl of anti-CD107a and 5 µg/ml brefeldin A (BioLegend) at an effector to target ratio of 1/5 in a 96 well plate in RP10 supplemented with 1 ng/ml IL-15 (Peprotec). After 5 hrs of incubation at 37 °C, 5% CO2, samples were washed and stained with anti-CD19-BV510, anti-CD3-PerCy5.5, anti-CD56-BUV395, anti-CD16-BV758, anti-KIR2DL1-FITC, anti-KIR2DS1/L1-PE (clone 11PB6) for 20 mn at room temperature. Cells were washed, fixed and flow cytometry analysis was performed on BD LSR Fortessa.

### Tetramer staining

HLA-C*06:02- SRGPVHHLL-PE tetramer was provided by the NIH Tetramer Core Facility and used for staining of target cell lines. Briefly, 2*10^5^ cells of each target cell lines were incubated on ice for 5 mn in 50 µL blocking buffer (sterile PBS+ 10% human serum+ 3% fetal bovine serum (FBS)) in a 96 well plate. Cells were washed and resuspended in 50 µL blocking buffer and the tetramer was added at a dilution of 1/100 for 60 mn on ice in the dark. The samples were washed with FACS buffer (PBS+ 3% FBS) and stained with the corresponding antibodies for 30 mn on ice on the dark. After two washing rounds with FACS buffer, samples were fixed in 4% paraformaldehyde and analyzed by flow cytometry (BD LSR Fortessa). Measurement of HLA-C*06:02- SRGPVHHLL-PE tetramer binding was assessed as MdFI of PE.

### Data acquisition, analysis and statistics

Flow cytometry data were analyzed using FlowJo software version 10.0.6 (Tree Star), and statistical analysis was performed using GraphPad Prism 5 (GraphPad Software). Each experiment was repeated independently 3 times except where stated otherwise. When indicated, statistical tests were performed assuming a non-parametric population using Kruskal-Wallis Test followed by post Dunn test analyzing all pairs of column. *^,^** and *** corresponded to p < 0.05; p < 0.01 and p < 0.001, respectively.

## Electronic supplementary material

Supplementrary figures
