# Supplementary material for: Peptide-specific engagement of the activating NK cell receptor KIR2DS1
Source: Sci Rep. 2017 May 25;7:2414. doi: 10.1038/s41598-017-02449-x (PMC5445099; doi:10.1038/s41598-017-02449-x)
Supplement: Supplementary file 1 — Supplementrary figures [file 41598_2017_2449_MOESM1_ESM.pdf]

Peptide-specific engagement of the activating NK cell receptor KIR2DS1

Anaïs Chapel[1], Wilfredo Garcia-Beltran[3], Angelique Hölzemer[2], Maja Ziegler[1], Sebastian Lunemann[1], Gloria Martrus[1], Marcus Altfeld[1]

1. Heinrich Pette Institute, Leibniz Institute for Experimental Virology, Hamburg, Germany;

2. Department of Internal Medicine, University Hospital Eppendorf (UKE), Hamburg, Germany;

3. Ragon Institute of MGH, MIT and Harvard, Cambridge, MA, USA

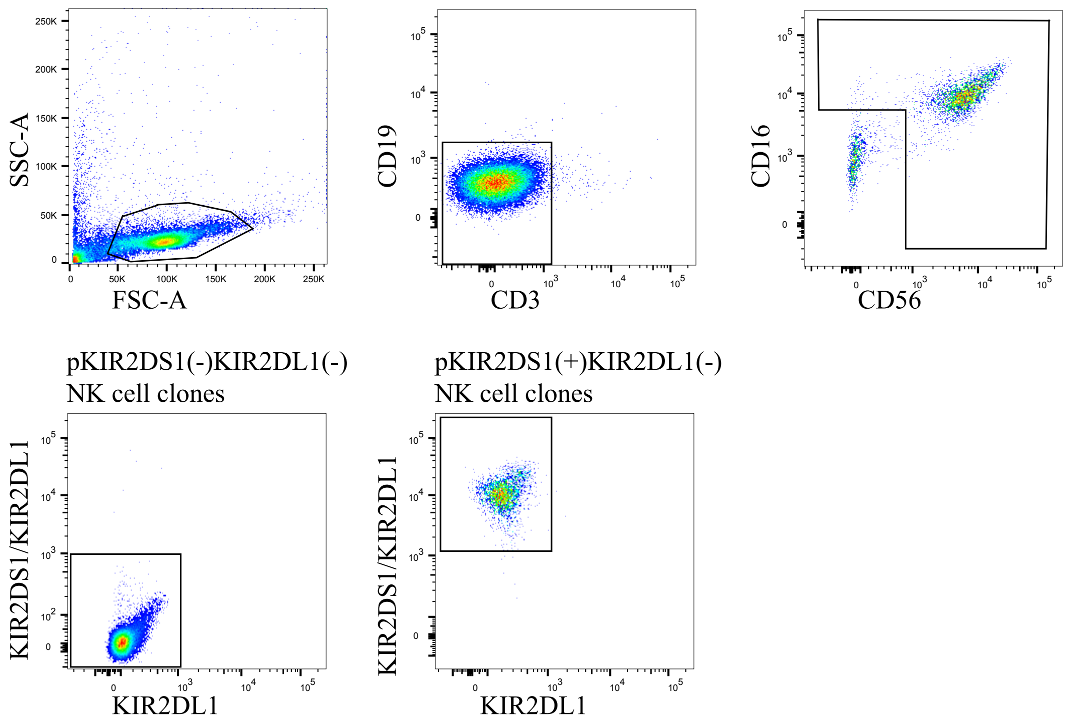

Supplementary Figure 1. Primary NK cell clones gating strategy. The NK cell clones were gated per size (SSC-A: Side Scatter Area; FSC-A: Forward Scatter Area), CD19 and CD3 as dumb channel, CD16 and CD56 expression and KIR2DS1+/- KIR2DL1- expression.

Peptide-specific engagement of the activating NK cell receptor KIR2DS1

Anaïs Chapel[1], Wilfredo Garcia-Beltran[3], Angelique Hölzemer[2], Maja Ziegler[1], Sebastian Lunemann[1], Gloria Martrus[1], Marcus Altfeld[1]

1. Heinrich Pette Institute, Leibniz Institute for Experimental Virology, Hamburg, Germany;

2. Department of Internal Medicine, University Hospital Eppendorf (UKE), Hamburg, Germany;

3. Ragon Institute of MGH, MIT and Harvard, Cambridge, MA, USA

Stabilizing peptides	Type and location	sequence
HLACW6-FL9	synthetic	F**R**PDLVSM**L**
HLACW6-YT9	synthetic	Y**R**PDTPHQ**I**
HLACW6-GV9	synthetic	G**R**MMVKIQA
HLACW6-SV9	synthetic	S**R**GPVHHL**L**
HLACW6-YH9	synthetic	Y**R**FNHGTLF
HLACW6-FQ9	synthetic	F**R**AWQAAL**V**
HLACW6-FE9	synthetic	F**R**SAEIKA**L**
HLACW6-MF9	synthetic	MTKPFTVD**L**
HLACW6-FG9	synthetic	F**R**MAGFMN**V**
HLACW6-YG9	synthetic	Y**R**MNGSSQ**I**
HLACW6-YA9	synthetic	Y**R**NQALIA**I**
HLACW6-FR9	synthetic	FGMERNYLF
HLACW6-FQ9	synthetic	F**R**AEQAYN**V**
HLACW6-FS9	synthetic	F**R**NISIRW**I**
p24 GAG-B142	HIV-1 p24 $GAG_{253-284}$	V**R**MYSPTSI**L**
p24 GAG-B172	HIV-1 p24 $GAG_{290-318}$	L**R**AEQASQE**V**
gp120-B51-OPT1	HIV-1 $gp120_{381-395}$	FFYCNTTQ**L**
RT-B24	HIV-1-$RT_{150-190}$	IFQSSMTKILEPFRK
RT-B24 –OPT1	HIV-1-$RT_{150-190}$	FQSSMTKI**L**
gp120-B1	HIV-1 $gp120_{272-288}$	NAKTIIVH**L**

Supplementary Table 1. 20 peptides stabilizing HLA-C*06:02 and their characteristics. Bold letter indicated the amino acid identified as binding motif for HLA-C*06:02 stabilization.

Peptide-specific engagement of the activating NK cell receptor KIR2DS1

Anaïs Chapel[1], Wilfredo Garcia-Beltran[3], Angelique Hölzemer[2], Maja Ziegler[1], Sebastian Lunemann[1], Gloria Martrus[1], Marcus Altfeld[1]

1. Heinrich Pette Institute, Leibniz Institute for Experimental Virology, Hamburg, Germany;

2. Department of Internal Medicine, University Hospital Eppendorf (UKE), Hamburg, Germany;

3. Ragon Institute of MGH, MIT and Harvard, Cambridge, MA, USA

Stabilizing peptides	sequence
**HLACW6-SV9**	**SRGPVHHLL**
R_2AL_9A	S**A**GPVHHL**A**
R_2A	S**A**GPVHHLL
L_9A	SRGPVHHL**A**
H_7A	SRGPVH**A**LL
H_7D	SRGPVH**D**LL
H_7G	SRGPVH**G**LL
H_7R	SRGPVH**R**LL
H_7S	SRGPVH**S**LL

Supplementary Table 2. Amino acid modifications of the peptide SRGPVHHLL. Bold letter indicated the amino acid change of the SRGPVHHLL peptide sequence.
